# A retrospective big data study using healthcare insurance claims to investigate the role of comorbidities in receiving low vision services

**DOI:** 10.3389/frhs.2024.1264838

**Published:** 2024-03-04

**Authors:** M. L. Stolwijk, R. M. A. van Nispen, S. L. van der Pas, G. H. M. B. van Rens

**Affiliations:** ^1^ Department of Ophthalmology, Amsterdam UMC, Vrije Universiteit, Amsterdam, Netherlands; ^2^ Quality of Care, Amsterdam Public Health, Amsterdam, Netherlands; ^3^ Department of Epidemiology and Data Science, Amsterdam UMC, Vrije Universiteit, Amsterdam, Netherlands; ^4^ Methodology, Amsterdam Public Health, Amsterdam, Netherlands

**Keywords:** low vision services, low vision rehabilitation, healthcare claims data, visual impairment, referral, mental comorbidity, physical comorbidity

## Abstract

**Introduction:**

The aim was to examine the association between physical and mental comorbidity with receiving low vision services (LVS).

**Methods:**

A retrospective study based on Dutch claims data of health insurers was performed. We retrieved data (2015–2018) of patients (≥18 years) with eye diseases causing severe vision loss who received LVS at Dutch rehabilitation organizations in 2018 (target group) and patients who did not receive LVS, but who received ophthalmic medical specialist care for glaucoma, macular, diabetic retinal and/or retinal diseases in 2018 (reference group). For examining the association between the patients' comorbidities and receiving LVS, multivariable logistic regression was used. The relative quality of five different models was assessed with the Akaike Information Criterion (AIC).

**Results:**

The study population consisted of 574,262 patients, of which 8,766 in the target group and 565,496 in the reference group. Physical comorbidity was found in 83% and 14% had mental comorbidity. After adjustment for all assumed confounders, both physical and mental comorbidity remained significantly associated with receiving LVS. In the adjusted model, which also included both comorbidity variables, the best relative quality was found to describe the association between mental and physical comorbidity and receiving LVS.

**Conclusions:**

Mental comorbidity seemed to be independently associated with receiving LVS, implying that the odds for receiving a LVS referral are higher in patients who are vulnerable to mental comorbidity. Physical comorbidity was independently associated, however, the association with receiving LVS might not be that meaningful in terms of policy implications. Providing mental healthcare interventions for people with VI seems warranted.

## Introduction

Low vision services (LVS) are essential in eye care. Through different kind of training and support, such as training in the use of low vision aids, computer training, orientation and mobility training and psychological support they offer people with a visual impairment (VI) the opportunity to participate in society and regain or maintain independence, contributing to a better quality of life.

Despite the relevance of LVS, research has repeatedly shown that access is jeopardized by barriers in the referral pathways towards LVS. However, the role of comorbidities of people with VI in receiving LVS is not fully understood. Although in some studies physical comorbidity or poorer health status has been identified as a barrier for LVS access, this has not been confirmed in other studies (1–4). In turn, it has been suggested that a great amount of people with VI utilizing LVS experience anxiety and/or depression, but in another study a hindering role of mental comorbidity in receiving LVS has been reported (3, 5). Differences in study outcomes may be explained by study design and limitations in data-analysis.

The aim of this study was to examine the association between physical comorbidity and mental comorbidity and receiving LVS, respectively, while accounting for potential confounders. As this will be the first study, to our knowledge, that examines the relationship between comorbidities and receiving LVS based on population-based healthcare claims data in a high-income country where LVS is fully funded and provided nationwide, we expect to find results that are specific for this healthcare context. Insights may be valuable for policy makers and healthcare providers to diminish barriers for vulnerable subgroups of people with VI in the referral pathways to LVS. This, in turn, ensures that more people in need receive the care they require.

## Methods

Administrative healthcare claims data between 2015 and 2018 were used of patients ≥18 years with eye diseases that cause severe vision loss who received care in Dutch outpatient LVS in 2018 (*n *= 8,766) and patients who did not receive LVS, but ophthalmic medical specialist care for glaucoma, macular, diabetic retinal, and/or retinal diseases in 2018 (*n *= 565,496). For both groups, patients who received LVS in 2015–2017 and thus, before 2018, were excluded to allow examination of the association with comorbidity and first time receipt of LVS in the four-year period. The data related to healthcare provided within the Dutch Health Insurance Act and was retrieved from Vektis C.V., a healthcare information center which routinely collects claims data of all Dutch health insurers (6).

The dependent variable was LVS utilization, which was defined as having received LVS at least once in 2018. Physical comorbidity was based on medical specialist care other than ophthalmology in 2015–2017, which was available as total costs per patient per medical specialty for each of the three years. Medical specialties related to diseases of the respiratory system, musculoskeletal system, cardiovascular system, skin and subcutaneous tissue, digestive system, urogenital system, other disorders of the nervous system and senses, endocrine/nutritional/metabolic diseases, epilepsy, hearing disorders/inner ear, multiple sclerosis, Parkinson's disease, allergies, infectious diseases, injury, neoplasms, and diseases of the blood and blood-forming organs. Having physical comorbidity was defined as having at least one cost registration within at least one of these medical specialties between 2015 and 2017. Mental comorbidity was based on the type of mental healthcare facility in which someone had been treated, encompassing basic and specialized mental healthcare, psychological care provided by general practice specialized mental healthcare nurses and other psychological care. It was also available as reimbursed costs per year per person. Mental comorbidity was defined as having at least one cost registration between 2015 and 2017. Based on relevance and availability, age, sex, socio-economic status (SES), area of residence and amount of ophthalmic diagnoses, were retrieved from the claims data and modeled as confounders. SES was defined as low, middle or high SES, and area of residence was categorized as urban and rural area of residence. Amount of ophthalmic diseases was determined by the amount of different diagnoses (glaucoma, macular, diabetic retinal, retinal, and/or other eye diseases), with the categorization into 0–1 diagnoses and 2 or more diagnoses.

We used multivariable logistic regression for examining the association between the patients' comorbidities and receiving LVS. Both the dependent variable (LVS utilization) and the determinants (comorbidities) were dichotomous. The potential confounders were selected according to the “disjunctive cause criterion”, as proposed by VanderWeele and Shpitser (7) whereby variables are classified as confounders if they can be considered causes of the determinant, the dependent variable or both. There were missing data for SES, area of residence and amount of ophthalmic diagnoses, which were assumed to be missing at random (MAR) and imputed according to Lanning (8). Furthermore, assumptions for logistic regression were tested, after which age was log transformed (9). We tested two crude models that only considered the association between the comorbidities and receiving LVS, and three models that included potential confounders. To identify the model with the best relative quality, we calculated the Akaike Information Criterion (AIC) for each model (10).

Data for this study was pseudonymized and aggregated to a minimum subgroup level of *n* > 10 to guarantee confidentially of individual patient's and care provider's information (11). The Medical Ethics Committee of Amsterdam University Medical Centers, location VUmc approved the study protocol.

## Results

Of the entire study population (*N *= 574,262), 83% had physical comorbidity (79.8% LVS-users vs. 83.3% LVS non-users) and 14% had mental comorbidity (18.6% LVS-users vs. 14.0% LVS non-users) (Table 1).

**Table 1:** Characteristics of the study population (*N *= 574,262).

	LVS users *n* = 8,766	LVS non-users *n* = 565,496	Total *N* = 574,262
	*n* (%)	*n* (%)	*N* (%)
Sex, female	4,636 (52.9)	303,354 (53.6)	307,990 (53.6)
Age, year, range 18–106, mean (SD)	65.27 (19.61)	69.68 (13.38)	69.61 (13.51)
Age groups			
18–29	578 (6.6)	7,011 (1.2)	7,589 (1.3)
30–39	497 (5.7)	10,334 (1.8)	10,831 (1.9)
40–49	799 (9.1)	27,156 (4.8)	27,955 (4.9)
50–59	1,306 (14.9)	67,572 (11.9)	68,878 (12)
60–69	1,364 (15.6)	131,822 (23.3)	133,186 (23.2)
70–79	1,635 (18.7)	186,352 (33.0)	187,987 (32.7)
80–89	1,910 (21.8)	115,823 (20.5)	117,733 (20.5)
90+	677 (7.7)	19,426 (3.7)	20,103 (3.5)
<65	3,863 (44.1)	167,565 (29.6)	171,428 (29.9)
≥65	4,903 (55.9)	397,931 (70.4)	402,834 (70.1)
Socio-economic status			
Missing	70 (0.8)	3,309 (0.6)	3,379 (0.6)
Low	3,259 (37.2)	206,214 (36.5)	209,473 (36.5)
Middle	3,280 (37.4)	213,275 (37.7)	216,555 (37.7)
High	2,157 (24.6)	142,698 (25.2)	144,855 (25.2)
Area of residence			
Missing	44 (0.5)	1,419 (0.3)	1,463 (0.3)
Urban	5,911 (67.4)	393,609 (69.6)	399,520 (69.6)
Rural	2,811 (32.1)	170,468 (30.1)	173,279 (30.2)
Amount of ophthalmic diagnoses^a^			
Missing	2,294 (26.2)	0 (0)	2,294 (0.4)
0–1	5,790 (66.1)	505,822 (89.4)	511,612 (89.1)
2 or more	682 (7.8)	59,674 (10.6)	60,356 (10.5)
Physical comorbidity			
Yes	6,998 (79.8)	471,097 (83.3)	478,095 (83.3)
No	1,768 (20.2)	94,399 (16.7)	96,167 (16.7)
Mental comorbidity			
Yes	1,634 (18.6)	78,976 (14.0)	80,610 (14.0)
No	7,132 (81.4)	486,520 (86.0)	493,652 (86.0)

Data are *n/n* (%) or *n/N* (%).

SD, standard deviation; LVS, low vision services.

^a^ Within ophthalmic medical specialist care based on the years 2015–2018. Amount of ophthalmic diagnoses of glaucoma, (diabetic) retinal or macular diseases. Having 0 ophthalmic diseases means that patients did not have one of these four diagnoses, but had another diagnosis. This only applies to the LVS-users, as the reference group was selected based on the four diagnoses.

In the crude models (1 and 2), both physical and mental comorbidity were significantly associated with receiving LVS (Table 2). Where physical comorbidity was negatively associated with receiving LVS, mental comorbidity was positively associated. After adjustment for confounders in model 3, 4 and 5, both determinants remained significantly associated with receiving LVS. However, there was a slight decrease in the estimates.

**Table 2:** Association between mental and physical comorbidity and receiving LVS**.**

Independent variables	Model 1^b^		Model 2^b^		Model 3^c^		Model 4^c^		Model 5^d^	
	OR [95%]	*p* ^a^	OR [95%]	*p* ^a^	OR [95%]	*p* ^a^	OR [95%]	*p* ^a^	OR [95%]	*p* ^a^
Physical comorbidity	**0.79 [0.75 − 0.84]**	**<.0001**			**0.86 [0.81 − 0.90]**	**<.0001**			**0.84 [0.80 − 0.89]**	**<** **.** **0001**
Mental comorbidity			**1.41 [1.34 − 1.49]**	**<.0001**			**1.27 [1.20 − 1.34]**	**<.0001**	**1.29 [1.22 − 1.37]**	**<** **.** **0001**
AIC	90,652.749		90,579.690		88,443.363		88,404.145		88,366.811	

OR, odds ratio; AIC, akaike information criterion.

^a^ Bold is significant at p < 0.05.

^b^ Unadjusted analyses.

^c^ Adjusted for sex (male, female), age (continuous), socio-economic status (low, middle, high), area of residence (rural, urban) and amount of ophthalmic diagnoses (0–1, 2 or more).

^d^ Adjusted for sex (male, female), age (continuous), socio-economic status (low, middle, high), area of residence (rural, urban) and amount of ophthalmic diagnoses (0–1, 2 or more) and leaving both determinants physical and mental comorbidity in the model.

Model 5 had the best relative quality to describe the association between mental and physical comorbidity and receiving LVS, respectively. After adjusting for assumed confounders, patients with physical comorbidity had a 0.84 lower odds of receiving LVS compared to patients without physical comorbidity. Patients with a mental comorbidity had a 1.29 higher odds of receiving LVS compared to patients with no mental comorbidity.

## Discussion

Our study showed that both, having mental comorbidity and physical comorbidity were independently associated with receiving LVS after adjusting for confounding factors.

Patients with a mental comorbidity had a 1.29 higher odds of receiving LVS compared to patients with no mental comorbidity, which contradicts earlier study results (3). Findings indicate that patients who are more vulnerable to mental comorbidity and who had mental complaints severe enough to warrant mental healthcare, have a greater chance of receiving LVS. Mental comorbidity may amplify patients' LVS needs and stimulate patients to discuss them with their provider, which in turn might facilitate referral to LVS. Mental comorbidity of patients might also be a trigger for referring healthcare providers, such as ophthalmologists and optometrists, to be more aware. However, LVS may not be necessarily provided for mental complaints but could also be given for practical support. Previous research found a prevalence of 32% of subthreshold depression and a prevalence of 16% of subthreshold anxiety in a LVS population of older adults (aged ≥60 years) in the Netherlands and Flanders (the Dutch speaking part of Belgium) (12). Another meta-analysis, which included studies from around the world, found a prevalence of approximately 25% of depression in patients with VI of eye clinics and low vision rehabilitation services. The majority of these patients were 65 years or older (13). These numbers differ from our study, where we found that 19% of the LVS-users had mental comorbidity. Since our study does not provide insight into patients who had mental complaints but did not receive mental healthcare, and included patients who were 18 years or older who could have various mental disorders, it is very likely that mental comorbidity in our study is underestimated. This indicates that a substantial number of patients do not receive mental healthcare, which stresses the importance for providing mental health interventions for people with VI. Literature further suggests that mental health complaints may be underdetected by eye care providers, such as ophthalmologists and optometrists (14, 15). Our findings imply that providers should be aware of mental comorbidity in patients, so that those in need of psychological support can receive that care.

Furthermore, patients with physical comorbidity had a 0.84 lower odds of receiving LVS compared to patients with no physical comorbidity. This is in line with previous literature (1–3). A possible explanation could be that patients with physical comorbidity may refuse referral, because of prioritizing other physical health problems and treatments. As a result, their LVS needs may be demoted to a secondary concern. Physical comorbidity might also affect patients' mobility and thus, LVS access. It should be noted however, that the vast majority of the study population had physical comorbidity, which might be explained by the fact that we selected a population that is more vulnerable to physical comorbidity by definition. Therefore, this determinant might not be that meaningful in terms of policy implications.

A strength of our study is the large sample size, which increases statistical power, and the use of population-based healthcare claims data of almost all Dutch citizens (99% of 17.2 million in 2018), which increases generalizability. Furthermore, to our knowledge, this is the first study on the association between physical and mental comorbidity with receiving LVS.

However, healthcare claims data are not initially intended for scientific purposes. In our study, valuable information about visual acuity, visual field defects and severity of the VI was unavailable, whereas earlier severity was found to be strongly related with receiving LVS (16).

Furthermore, we could not differentiate between specific types of mental diagnoses, because this information was not available in the data. It would have been interesting to get more insight into which mental comorbidities exactly are associated with receiving LVS as this information is missing from literature. Furthermore, in another qualitative study on LVS access, mental comorbidity, which was identified as a barrier, included anxiety and/or depression (3). Our study included all types of mental diagnoses and study results therefore might differ. The fact that comorbidity could also be based on one-off appointments as it was defined having utilized the certain types of healthcare at least once within the period of three years, might have limited our results as well. Consequently, the 83% of patients having physical comorbidity might have been an overestimation.

Besides that, our results might have been affected by coding errors as this is a common bias in administrative claims data (17). This may have introduced selection bias, possibly affecting generalizability of our results. There are contradicting results regarding validity of Dutch healthcare claims data and validity studies on Dutch ophthalmic healthcare claims data are missing (18, 19).

Moreover, although we described a large group of patients who received LVS (*n *= 8,766), it was relatively small compared to the reference group (*n *= 565,496). We may have introduced selection bias by comparing this relatively small group of patients with all kinds of eye diseases with a large group with a selection of eye diseases that are most likely to cause VI.

Lastly, our results may not be generalizable to other countries with other healthcare systems and LVS referral procedures.

Our findings demonstrate that mental comorbidity has an independent positive association with receiving LVS. This indicates that that the odds for receiving a LVS referral are higher in patients who are vulnerable to mental comorbidity, hence, having mental comorbidity seems to be a facilitator in the referral pathway towards LVS. Physical comorbidity seemed to be negatively associated with receiving LVS, however this association might not be that meaningful for policy makers as the majority of the study population had physical comorbidity and we selected a population that is more vulnerable to physical comorbidity by definition. Future research should investigate the influence of other potential confounders. Finally, as it is likely that the prevalence of mental comorbidity in our study is underestimated, researchers and policy makers should be aware of mental complaints in patients and focus on providing mental healthcare interventions for people with VI.

## Data Availability

The data analyzed in this study is subject to the following licenses/restrictions: Data are not available due to European privacy regulations. Information requests about access of the data can be directed to MS, m.stolwijk@amsterdamumc.nl.
